# Monthly Summary

**Published:** 1882-03

**Authors:** 


### MONTHLY SUMMARY.

*A Tooth in a Bronchus for five days Causes Pneumonia.*—Mrs. K——, aged thirty-five; healthy; wife of a farmer.

August 19th.—She had thirteen teeth extracted from the upper jaw at one sitting, by an experienced (?) dentist in New York City. Upon discontinuing the administration of gas (nitrous oxide) she fainted. Then she was seized with severe epigastric pain, urgent dyspnœa, cough, nausea, and prostration. It was several hours before she could be removed to her home in Orange.

August 22d.—The dyspnœa, nausea, and cough continued, and in addition there was severe pain in the left axillary line on a level with the nipples, at which place there was a zone of bronchial breathing and voice, and dullness over a space not larger than a silver dollar. In the neighborhood were mucous râles. This consolidation was preceded for two days by pulmonary congestion. The cough to-day less in frequency and severity. Sputa scanty, serous, and muco-purulent, streaked with blood. Temperature, 102° F.; respiration, 40; pulse, 102.

August 23d.—Temperature, 104° F.; respiration, 38; pulse, 110.

August 24th (sixth day.)—At 9 A. M. there was a severe coughing with urgent dyspnœa (the patient says "she thought she would be suffocated,") terminating with a spasm and vomiting, after which relief was obtained. In the matter coughed up and vomited was found a second molar tooth. It was possitively certain that it did not come from either jaw at this time, as both had been carefully examined since the illness began. Blood was abundant in the sputa, which was muco-purulent in character. Cough very much easier. The whole lower lobe gave bronchial breathing and voice, with dullness. Temperature, 104° F.; respiration, 44; pulse, 124 (two hours after the coughing struggle.)

August 25th, (seventh day.)—Patient generally easier. Sputa copious and blood-streaked. Temperature, 100.5° F.; respiration, 32, pulse, 100.

August 28th.—Prostration marked. Copious sweats. Blood disappeared from sputa.

September 19th (one month.)—Patient up and about. Vesicular respiration returned to lung. Râles abundant.

September 25th.—Patient well, except weak.

A gentleman of large experience in extracting teeth says that, "even when the greatest care is exercised, a tooth will sometimes slip out of the forceps. If it should happen to fall into the throat it will be swallowed. No exercise of skill can prevent this. When it is swallowed it does no harm. If it lodges in the throat—which is a very rare occurrence—it can be thrown out by an emetic." Just so, but in the case cited a whole molar tooth, perfectly sound (heaven forgive the dentist for pulling such a good tooth!) went past the throat, through the glottis to the lung. Here if it had not been dislodged and coughed out, abscess and almost surely death would have been the result.

Now, where so many teeth are extracted at one sitting, the operator must, of necessity, be expeditious; but it does seem as if there should be time enough to remove entirely from the mouth any tooth or part of a tooth loosened from its bed in the jaw. The person at this time is in a state of induced coma, the sensibility of the larnyx is very much less than normal, so that resistance to the entrance of a foreign body past the glottis is likewise diminished.

Thus it behooves operators to use a care in avoiding this serious accident. Where a patient fails to recover upon discontinuing the administration of gas, every loosened tooth or part of a tooth should be accounted for, and means be employed at once to discover and release the foreign body if it be caught anywhere in the throat or respiratory tract.—*T. R. Chambers, M. D.*

---

*Annealing Gold.*—Making gold cohesive over the flame of a lamp while filling teeth is bad practice. It is impossible to make it uniformly cohesive, and in many parts is sure to be so stiff and unyielding as to make imperfect fillings. They will stay, and finish up nicely; but after a year or two it will be proved that many of them never were tight fillings. They are leaky, full of pockets, and not well impacted against the walls cf the cavity. Besides, there is no necessity of this extra work at the chair; gold can be made cohesive much better by the manufacturer. The notion with some dentists that it is better when done immediately before using, is fallacious. When once made cohesive, gold remains so for a long time even when exposed to the air. If it is desirable to produce this quality in gold, it is much better to heat it on mica over a fire which will reach all parts alike. But even this produces cohesiveness at the expense of other good qualities. It is a very delicate and difficult matter to make gold cohesive and yet have it remain soft and velvety. Formerly it seemed impossible. Now many makes approach these desirable qualities in combination.—*Items of Interest.*

---

*An Anæsthetic Car.*—A correspondent of the *British Medical Journal* gives the following description of Bert's anæsthetic car for giving nitrous oxide and compressed air. "I was at the St. Louis Hospital one morning; the car was there, and some operations were to be done in it. We—the patient, doctor, and his students—went into the car; the door, air-tight, was closed, and air forced into the car; in a few minutes my ears began to feel strange, and I was told to swallow, yawn, and blow my nose, which I did every few minutes, and so made the pressure equal on both sides of the drums of my ears. The patient laid himself down on the operating-table, and the anæsthetic agent was given him. He took it very quietly, did not struggle, and was soon insensible. Whilst he was unconscious, an epithelioma was removed from his lower lip; after the wound was sewn up, the compressed air was allowed to escape; the patient got up from the table, walked out of the car, and laid down on the grass; he complained of no headache nor nausea, but said he felt just as usual. We also were glad to escape from the car, on account of the heat, which was very intense. During the operation, all the assistants and the surgeons pulled off their coats and waistcoats, and yet they perspired very freely. The car is on wheels, and is carried about from hospital to hospital; the hospitals being under Government, the car is a public one, and is taken all over Paris."—*Med. Record.*

---

*Keeping Warm.*—Some Scientific Facts and Principles, Plainly Stated, with Practical Lessons they Teach:

Under the above heading the *American Agriculturist* for January gives a lengthy and very practical article, which should be generally read. We make the following brief extracts:

All human beings are so constituted that while some parts may be temporarily benumbed with cold without danger, the blood that circulates through the heart and through the system generally, must be maintained uniformly at just about 98½° of temperature (98½° Fahrenheit, or 37° centigrade.) If the heat of the blood rises or falls only six or seven degrees from this normal point, and continues so, fatal results are expected. Every degree that disease elevates the general internal heat about 100°, is a rapid approach towards the danger point, and when only 105° is reached, the most skillful efforts of physicians are usually needed to save life. On the contrary, even in the coldest regions where the thermometer marks 60° or 70°, or more, below the freezing point, the internal heat must be kept up, and if it falls only from 98° to 94° or 93°, and continues thus, there is great danger that the wheels of life will stop. (These are general statements, applicable to a healthy condition. Cases have been reported where in tetanus or lock-jaw the temperature rose to nearly 111°, while in asthma it sunk to 78°, and in cholera to 67°.)

When the oxygen of the air unites with the carbon of hard coal or charcoal, or of wood, or of flour, meat, or of any other food, a compound is formed, viz., a gas, which we call Carbonic Acid. The process of forming this compound sets at liberty heat which was before entirely concealed, or which existed in another form, and when coal or wood is burned rapidly we have a hot fire. The same process goes on when wood rots away, but the heat is developed so slowly that we do not notice it.

Precisely the same thing is taking place in the human body all the time. The food we eat and digest, is in part absorbed into the blood, and carried by it everywhere throughout the body. But at the same time the blood passing through the lungs is constantly picking up oxygen there from the air which we breathe into the lungs, and this, too, goes all through the body, and at millions of points one carbon atom of the food is uniting with two atoms of oxygen from the air, forming carbonic acid, and setting heat at liberty, precisely the same as takes place in burning wood or coal in the stove. There is only a small product of heat at any one point in the blood, but it takes place at so many points that there is enough developed to keep up the general temperature. And a wonderful provision it is, that without our supervision, or knowledge even, this ever burning fire goes on within us, just so as to keep the whole body at about 98°. (We speak only of the main source of animal heat. There are other combinations going on in the body, which produce more or less heat, such as the union of oxygen, which escapes as water; the union of minute quantities of sulphur and of phosphorus with oxygen, etc. Most probably more or less heat is also derived from the mechanical movements of the various organs.)

If the supply of food fails in the blood for a time, from fasting or sickness, then the oxygen in the blood attacks any stored up fuel, as fat, flesh and other organs of the body, using their carbon to keep up the ever necessary warmth. The weight of the body grows less, and when no more fat or flesh can be found to make heat, cold and death come on. The same result follows if the lungs become so diseased as not to furnish the air supplying oxygen fast enough to keep up the internal fire. Stop the entrance of air for a few minutes by closing the windpipe with a cord, or by filling the lungs with water, and heat production stops in the blood, the temperature falls below 93°, and the human machinery ceases to work.

The carbonic acid produced is poisonous. In the stove it escapes through the pipe. That formed in the blood is carried to the lungs and thrown out into the air. Too many persons breathing in a close room fill it with so much carbonic acid that it becomes very unhealthful, if not dangerous.

The blood carries the heat to the surface of the body, as well as to all other parts, and a good deal of heat escapes off into the air. If the air is cold this escape is more rapid, and more heat must be produced within to supply the waste. That means more fuel, that is more food, or more flesh is consumed. Remember that the blood must be kept up to about 98°. Of course, then, in cold weather more food is necessary or the body becomes emaciated. But anything that stops escape of heat from the surface of the body, saves food, or saves using up flesh. Warm clothing, warm air, warm dwellings, warm stables, warm sheds, all help to stop this waste of heat.—*Am. Agriculturist.*

---

*Nerve Regeneration.*—It is really singular how much the nerves may be mutilated and broken, and yet ultimately recover. A friend of mine, in one of the most bloody engagements of the late war, received a bullet wound in his left arm. The bullet entered about two inches below the axilla, and passed through near the side of the bone, cutting the median nerve entirely off. The hand was paralyzed and bent inward. The wound was very painful and a long time in healing. For several years the arm was useless. At length he found it strengthening, the pain and tenderness disappearing, so that he could do something with it by way of assisting his right hand. After about four years it acquired a good degree of usefulness. At this time it is nearly fully restored. One could hardly have predicted so complete recovery after so destructive an accident. —*E. Chenery, M. D.*

